# Fluid damping of cylindrical liquid storage tanks

**DOI:** 10.1186/s40064-015-1302-2

**Published:** 2015-09-17

**Authors:** Joerg Habenberger

**Affiliations:** Basler & Hofmann AG, Forchstrasse 395, 8032 Zurich, Switzerland

**Keywords:** Fluid damping, Earthquake, Cylindrical liquid storage tanks

## Abstract

A method is proposed in order to calculate the damping effects of viscous fluids in liquid storage tanks subjected to earthquakes. The potential equation of an ideal fluid can satisfy only the boundary conditions normal to the surface of the liquid. To satisfy also the tangential interaction conditions between liquid and tank wall and tank bottom, the potential flow is superimposed by a one-dimensional shear flow. The shear flow in this boundary layer yields to a decrease of the mechanical energy of the shell-liquid-system. A damping factor is derived from the mean value of the energy dissipation in time. Depending on shell geometry and fluid viscosity, modal damping ratios are calculated for the convective component.

## Background

The dynamic behavior of liquid storage tanks and of structures in general is highly influenced by the structural damping. In engineering, an ideal fluid is usually assumed in the realm of dynamic analysis of liquid storage tanks. In doing this, the potential equation of the liquid may be divided into two decoupled components: (1) the impulsive component which describes the interaction of the liquid and the shell and (2) the sloshing motion of the free liquid surface which may be accounted for by the convective component.

After the modal decomposition of both components, a viscous damping is introduced to consider the dissipation of mechanical energy. The damping influences the resulting pressures as well as the amplitude of the convective fluid motion. If the response spectra method is used to calculate the dynamic response of the tank-liquid-system the spectral acceleration is determined directly by the damping ratios.

The damping of the impulsive component is mainly affected by the damping of the shell, and the fluid damping may be neglected. Depending on the material of the shell, damping ratios between 2 % (steel) and 5 % (reinforced concrete) are suggested for the Serviceability Limit State (Eurocode 8, Part 4 2006 or Scharf 1989). The damping ratios are larger for the Ultimate Limit State (4 % for steel and 7 % for concrete structures). These are typical and well established damping ratios (Stevenson 1980).

For the convective component damping ratios from 0 up to 5 % are proposed. The second draft of Eurocode 8, Part 4, e.g., suggests a damping ratio of 0.5 % “for water and other liquids”. Scharf (1989) recommends a value of 0 % independent of the content. It is also important to mention that there are no experimental or theoretical justifications of the proposed damping values concerning the sloshing oscillation and they are more or less “best guesses”.

With the potential equation of the ideal fluid only boundary and interaction conditions normal to the surface of the fluid can be satisfied. It is not possible to describe the adhesion of a real fluid to the tank wall and bottom by the potential equation. To fulfill the boundary conditions though a one-dimensional shear flow is superimposed on the potential flow of the ideal fluid. For this purpose at first the Navier–Stokes-equation is applied and simplified with respect to the conditions at the boundary layer of the fluid. A solution of the simplified form of the Navier–Stokes-equation is derived which describes the velocity in the boundary layer.

**Fig. 1:**
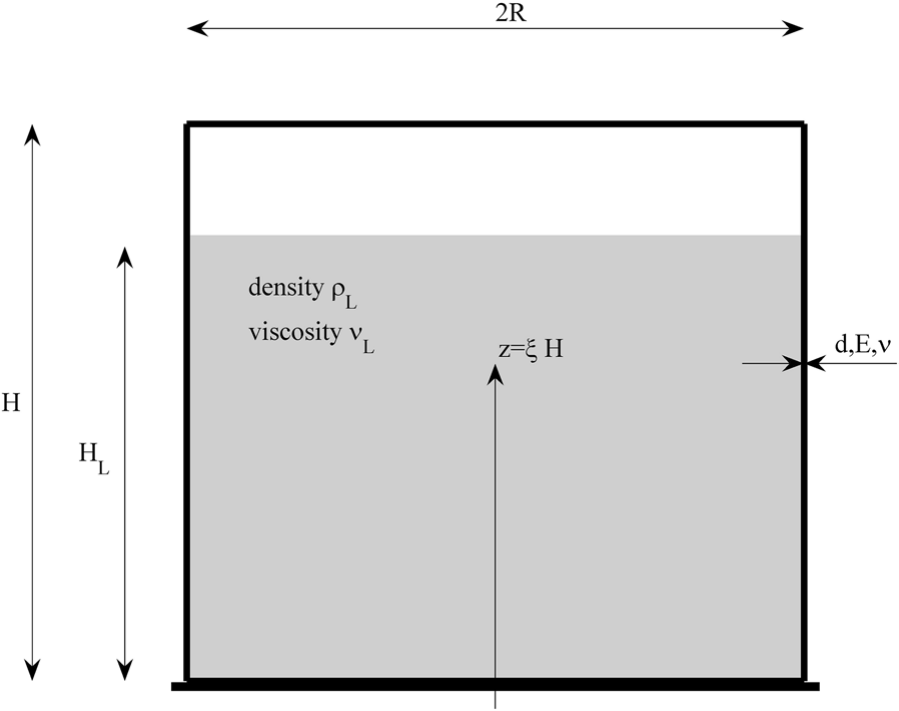
Definition of material and geometry parameters of the investigated vertical cylindrical liquid storage tanks: *R* radius, *H* tank height, *H*_*L*_ liquid height, *d*/*E*/$\nu$ thickness, Young’s-modulus and Poisson ratio of the tank shell

The shear flow in the boundary layer leads to a dissipation of mechanical energy. This energy dissipation is related to the damping ratio of the fluid oscillation. Damping ratios for the sloshing component are derived for different shell geometries and fluid viscosities. In Fig. 1 a cross-section of the investigated liquid storage tank with the corresponding material and geometry parameters is shown.

## Damping effects of a viscous fluid

### Equations of the incompressible, viscous fluid

The fluid is assumed to be incompressible and viscous. The friction pressures are proportional to the velocity of the liquid (Newton’s fluid with kinematic viscosity $\nu$). The condition of incompressibility (with the velocity field of the fluid ${\mathbf{v}} = (v_{\zeta } v_{\varphi } v_{\xi } )^{T}$) reads as follows (Sommerfeld 1988):

1 $$ \nabla \cdot{\mathbf{v}} = 0. $$

The Navier–Stokes-Equation without consideration of volume forces is:

2 $$ \frac{{\partial {\mathbf{v}}}}{\partial t} + ({\mathbf{v}}\nabla ){\mathbf{v}} = \nu_{L} \Delta {\mathbf{v}} - \frac{1}{{{\varrho }_{L} }}\nabla p. $$

Under the assumption of small oscillation amplitudes, the contribution of the nonlinear expression $({\mathbf{v}}\nabla ){\mathbf{v}}$ in (Eq. 2) can be neglected:

3 $$ \frac{{\partial {\mathbf{v}}}}{\partial t} = \nu_{L} \Delta {\mathbf{v}} - \frac{1}{{{\varrho }_{L} }}\nabla p. $$

By using the rotation of the velocity field $\omega = \nabla \times {\mathbf{v}}$ it is possible to transform the Navier–Stokes-Equation into a simpler form (Landau and Lifschitz 1987):

4 $$ \frac{\partial \omega }{\partial t} = \nu_{L} \Delta \omega . $$

According to Eq. (4), the rotation of the velocity field $\omega$ corresponds to the heat equation. Hence, there is a convective transport of the velocity vortices from the surface into the liquid. This process decays exponentially into the interior of the fluid. It is not possible for conservative forces to produce velocity vortices in a viscous fluid. There must be forces which can not be derived from a potential (Schaefer 1950). In the present case these are shear forces (frictional pressures) occurring at the tank wall. Concerning the oscillation of storage tanks, vortices are produced at the tank wall due to frictional pressure. The velocity vortices decrease exponentially into the interior of the liquid. Because of the exponential damping, the rotational flow occurs practically only in a small layer at the tank wall. The main part of the liquid is an irrotational flow and can be described by the following equation:

5 $$ \nabla \times {\mathbf{v}} = 0 \, \nabla \cdot{\mathbf{v}} = 0. $$

As derived from Eq. (5), one can see that $\Delta {\mathbf{v}} = 0$ (the Navier–Stokes-equation becomes the potential equation). Thus, the liquid behavior is everywhere in such a tank like this of an ideal (incompressible and frictionless) fluid. Only in a thin layer on the tank wall the potential flow is disturbed. The boundary conditions of a viscous liquid require the consistency of the liquid velocity at the surface and the velocity of the boundary (tank wall and bottom). With the equation of the ideal fluid, the boundary conditions normal to the free liquid surface and the tank wall and bottom can be satisfied. Hence, the normal component of the liquid velocity suffers only slightly from the rotational flow in the thin layer at the boundaries.

The Eq. (5) of the ideal fluid can not satisfy the boundary conditions concerning the consistency of the tangential fluid velocity and the velocity of the boundaries (tank wall and bottom, surface). The solution of the potential Eq. (5) gives tangential fluid velocities at the boundary different from those of the boundary itself. Thus, a significant change of the tangential velocity must occur across the thin boundary layer.

In order to investigate the characteristics and properties of the boundary layer, the simple one-dimensional shear flow of a viscous fluid over an oscillating plane is analyzed (Landau and Lifschitz 1987).

### Solution of the one-dimensional shear flow

**Fig. 2:**
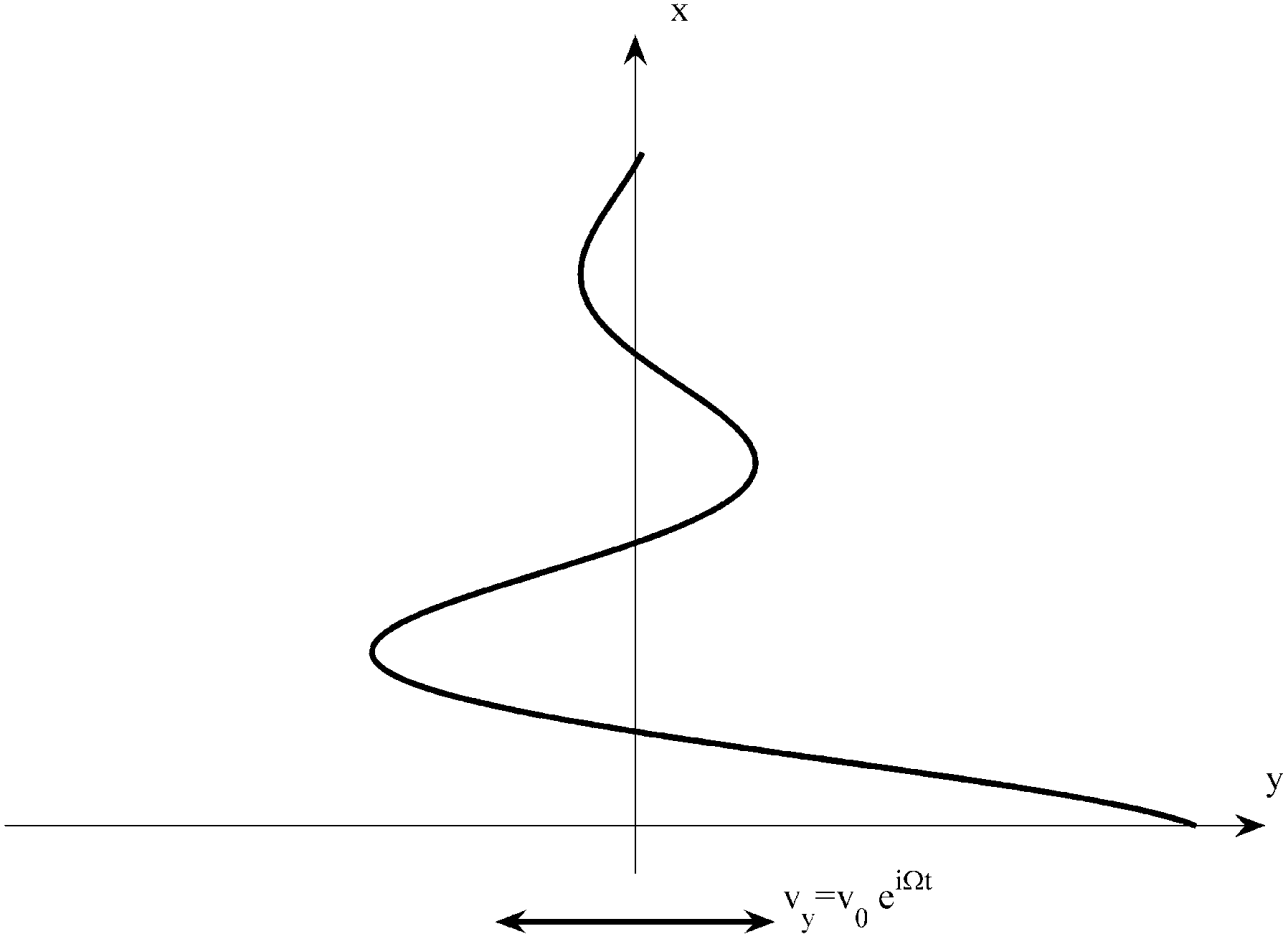
Decreasing shear flow of a viscous fluid above an oscillating yz plane with amplitude v_0_ and frequency Ω

An incompressible and viscous fluid located above the $xy$ Cartesian-plane ($x > 0$) is considered as next. The plane fulfills harmonic oscillations with the frequency ${{\Omega }}$ in $y$ direction. The velocity of the plane is given by $v = v_{0} e^{i\Omega t}$ (see Fig. 2).

Because of the symmetry of the one-dimensional shear flow all parameters depend only on time $t$ and the space coordinate $x$. Thus, the condition of incompressibility (Eq. 1) can be determined by the following expression:

6 $$ \frac{{\partial v_{x} }}{\partial x} = 0. $$

The outcome of this equation determines that $v_{x} = const$. On the $yz$-plane the boundary condition $v_{x} = 0$ at $x = 0$ must be considered. Hence, $v_{x}$ becomes $0$ for all $x$. All fluid velocities depend only on $x$ and $t$. Consequentially the expression $({\mathbf{v}}\nabla ){\mathbf{v}}$ can be simplified to $v_{x} \frac{\partial }{\partial x}{\mathbf{v}}$, and it becomes 0 because of the condition $v_{x} = 0$.

The Eq. (2) is transformed to the following:

7 $$ \frac{{\partial {\mathbf{v}}}}{\partial t} = - \frac{1}{{{\varrho }_{L} }}\nabla p + \nu_{L} \Delta {\mathbf{v}}. $$

The component in $x$ direction of Eq. (7) is:

8 $$ \frac{\partial p}{\partial x} = 0 $$

therefore, $p = const$. From symmetrical conditions it follows that $v_{z} = 0$. For this reason the component in the $y$ direction of Eq. (8) becomes:

9 $$ \frac{{\partial v_{y} }}{\partial t} = \nu_{L} \frac{{\partial^{2} v_{y} }}{{\partial x^{2} }}. $$

This expression is equal to the one-dimensional heat equation. It can be solved by the method of separation of variables with the approach $v_{y} (x,t) = f(x)e^{i\Omega t}$. Equation (9) becomes:

10 $$ i\frac{\Omega }{{\nu_{L} }}f - \frac{{{\text{d}}^{2} f}}{{{\text{d}}x^{2} }} = 0. $$

The general solution of Eq. (10) is:

11 $$ f(x) = C_{1} e^{{ik_{1} x}} + C_{2} e^{{ik_{2} x}} $$

with:

12 $$ k_{1,2} = \pm \sqrt {\frac{\Omega }{{2\nu_{L} }}} (1 - i). $$

The parameters $C_{1}$ and $C_{2}$ are determined by the boundary conditions. For $x \to \infty$ the velocity $v_{y}$ have to be finite: $C_{1} = 0$. The parameter $C_{2}$ is determined by the boundary condition $v_{y} (x,t)|_{x = 0} = v_{0}$:

13 $$ C_{2} = v_{0} . $$

Subsequently, the solution for the velocity in y direction is:

14 $$ v_{y} = v_{0} e^{{ - \frac{x}{{\delta_{L} }}}} e^{{i(\Omega t - \frac{x}{{\delta_{L} }})}} = v_{0} e^{i\Omega t} e^{{ - (1 + i)\frac{x}{{\delta_{L} }}}} . $$

The obtained expression describes a propagating harmonic wave. The wave amplitude decreases exponentially with the distance in x-direction. Thus, the solution describes a spatially damped, transversal wave ($v_{y}$ is perpendicular to the direction of propagation). The imaginary part of the complex wave number $k_{2}$ determines the damping parameter. In contrast, the real part of $k_{2}$ is inversely proportional to the wavelength ($\lambda = \frac{2\pi }{{\Re (k_{2} )}}$). The penetration depth $\delta_{L} = \sqrt {\frac{{2\nu_{L} }}{\Omega }}$ shows the decay of the damped oscillation into the fluid. The oscillation amplitude diminishes with the factor $e = 2.718 \ldots$ at about a distance of $x = \delta_{L}$. At a wavelength of approximately ($2\pi \delta_{L}$), the amplitude decreases with the factor $e^{2\pi } \approx 535$. The damping of the oscillation amplitude increases with the increase of the frequency ${{\Omega }}$ and is inversely proportional to the viscosity of the fluid. In contrast, the penetration depth decreases with increasing frequency and is directly proportional to the viscosity of the fluid.

### Tangential fluid velocity on the boundaries to the tank shell

The dimensions of the investigated storage tanks ($R$ and $H_{L}$) are large compared to the penetration depth $\delta_{L}$ and to the oscillation amplitudes (designated with $q$):

15 $$ R \gg \delta_{L} {\text{ and }}q \ll R. $$

There are no restrictions to the Reynold’ number. The neglecting of the term $({\mathbf{v}}\nabla ){\mathbf{v}}$ of the Navier–Stokes-equation (Eq. 2) stems from the following considerations. The operator $({\mathbf{v}}\nabla )$ describes the derivation in the direction of the fluid velocities. On the fluid boundaries, the largest velocities are those which are parallel to the surface. Along these lines a considerable change in velocity occurs only over distances with a length comparable to the dimensions of the tank ($R$ and $H_{L}$). According to this, the following relations are true: $({\mathbf{v}}\nabla ){\mathbf{v}}\sim \frac{{{\mathbf{v}}^{2} }}{R}\sim \frac{{q^{2} \Omega^{2} }}{R}$. The velocity has a magnitude of $v\sim q\Omega$. Thus, the magnitude of the partial derivative of the velocity $\frac{{\partial {\mathbf{v}}}}{\partial t}$ can be given $v\Omega \sim q\Omega^{2}$. A comparison of both relations shows that $({\mathbf{v}}\nabla ){\mathbf{v}} \ll \frac{{\partial {\mathbf{v}}}}{\partial t}$ actually holds for $q \ll R$.

Now a small section of the fluid surface is considered to determine the distribution of the tangential velocity. The dimensions of the surface section are small compared to the dimensions of the tank and large compared to the penetration depth $\delta_{L}$. By doing this, the surface section may be assumed to be a plane surface, and the results developed in the previous section can be used.

With $v_{y}$ the tangential velocity of the surface section is designated. On the surface sections ($x = 0$) the tangential velocities have to become 0 (adhesion condition of the viscous fluid). From the solution of the potential equation of the liquid (Eq. 5), a tangential velocity $v_{0} e^{i\Omega t}$ relative to the boundary at $x = 0$ is calculated:

16 $$ v_{y} = v_{0} e^{i\Omega t} \left[ {1 - e^{{ - (1 + i)\frac{x}{{\delta_{L} }}}} } \right]. $$

The $x$ axis points toward the normal of the surface.

### Damping effect of the viscous fluid

The assumption is made that the mechanical energy decreases in time with $E_{mech} = {\text{const}}\:e^{ - 2\gamma t}$. The damping factor $\gamma$ can be calculated by the following equation using the time-based mean value of the mechanical energy dissipation $\bar{\dot{E}}_{mech}$:

17 $$ \gamma = \frac{{\left| {\bar{\dot{E}}_{mech} } \right|}}{{2E_{mech} }}. $$

The mechanical energy is proportional to the square of the oscillation amplitude. Therefore, the decrease of the oscillation amplitude is specified by the factor $e^{ - \gamma t}$. The relation between the damping factor and the damping ratio is given by $\kappa = \frac{\gamma }{\omega }$. The time-based mean value of the mechanical energy dissipation is given in Eq. (18):

18 $$ \bar{\dot{E}}_{mech} = - \frac{1}{2}\sqrt {\frac{{\mu_{L} {\varrho }_{L} \Omega }}{2}} \int_{A} {\left| {v_{0} } \right|^{2} } {\text{d}}A. $$

If we assume small oscillation amplitudes, the mean values of the kinetic and potential energy are equal. The mechanical energy is double the kinetic and potential energy, respectively ($E_{mech} = 2\bar{E}_{kin}$). The kinetic energy of the fluid can be calculated by the equation:

19 $$ E_{kin} = \frac{{{\varrho }_{L} }}{2}\int_{V} {v^{2} } {\text{d}}V. $$

It is possible to transform the volume integral in an integral over the fluid surface by using the velocity potential $\varPhi$:

20 $$ E_{kin} = - \frac{{{\varrho }_{L} }}{2}\int_{A} \varPhi \frac{\partial \varPhi }{\partial n}{\text{d}}A $$

The expression $\frac{\partial \varPhi }{\partial n}$ indicates the velocity normal to the surface of the liquid (positive inside). The impulsive pressure on the surface is denoted by ${\varrho }_{L} \varPhi$.

## Damping of the convective component

The fluid potential of the harmonic sloshing oscillation in the nth axial and the 1st circumferential mode can be expressed by:

21 $$ \varPhi_{1,n} = qe^{i\Omega t} J_{1} (\lambda_{n} \zeta )\cosh (\lambda_{n} \alpha_{L} \xi )\cos \varphi $$

with the amplitude $q$ of the time harmonic function $e^{i\Omega t}$. Due to the assumed rigid tank walls on the free liquid surface, only fluid velocities normal to the boundary can occur. The kinetic energy is calculated using the following Eq. (22):

22 $$ E_{kin} = \frac{{{\varrho }_{L} }}{2}R^{2} \int\limits_{0}^{1} {\int\limits_{0}^{2\pi } \varPhi } \frac{\partial \varPhi }{\partial \xi }\zeta {\text{d}}\zeta {\text{d}}\varphi . $$

The mechanical energy of the sloshing oscillation in the nth axial and the 1st circumferential mode is calculated by the following Eq. (23):

23 $$ E_{mech,1,n} = q^{2} {\varrho }_{L} R\frac{\pi }{4}\cosh (\lambda_{n} \alpha_{L} )\sinh (\lambda_{n} \alpha_{L} )\frac{{\lambda_{n}^{2} - 1}}{{\lambda_{n} }}. $$

The main part of the energy dissipation results from the friction on the tank wall and bottom. The friction due to the rotational flow on the liquid surface is small and can be neglected. The dissipated energy arises from the friction on the tank wall and tank bottom due to the fluid flow in radial and circumferential direction.

The components of the fluid velocities can be calculated using the following relations:

- Velocity in axial direction on the tank wall:

24 $$ v_{\xi } = \frac{1}{H}\frac{{\partial \varPhi_{1,n} }}{\partial \xi }|_{\zeta = 1} = qe^{i\Omega t} \frac{1}{H}J_{1} (\lambda_{n} )\sinh (\lambda_{n} \alpha_{L} \xi )\lambda_{n} \alpha_{L} \cos \varphi . $$

- Velocity in circumferential direction on the tank wall:

25 $$ v_{\varphi } = \frac{1}{r}\frac{{\partial \varPhi_{1,n} }}{\partial \varphi }|_{\zeta = 1} = - qe^{i\Omega t} \frac{1}{R}J_{1} (\lambda_{n} )\cosh (\lambda_{n} \alpha_{L} \xi )\sin \varphi . $$

- Velocity in circumferential direction on the tank bottom:

26 $$ v_{\varphi } = \frac{1}{r}\frac{{\partial \varPhi_{1,n} }}{\partial \varphi }|_{\xi = 0} = - qe^{i\Omega t} J_{1} (\lambda_{n} \zeta )\frac{1}{\zeta R}\sin \varphi . $$

- Velocity in radial direction on the tank bottom:

27 $$ v_{\zeta } = \frac{1}{R}\frac{{\partial \varPhi_{1,n} }}{\partial \zeta }|_{\xi = 0} = qe^{i\Omega t} \frac{{\lambda_{n} }}{R}\left[ {J_{0} (\lambda_{n} \zeta ) - \frac{1}{{\lambda_{n} \zeta }}J_{1} (\lambda_{n} \zeta )} \right]\cos \varphi $$

with $I_{0}$ the modified Bessel functions of the 2nd kind and 0th order and by $J_{m} (y)$ the Bessel function of the 1st kind and order m with argument $y$ are designated. The parameters $\lambda_{n}$ are the roots of the equation: ${\text{d}}J_{1} (y)/{\text{d}}y = 0$. It has the solutions $\lambda_{1} = 1.8412,\;\lambda_{2} = 5.3314 \ldots$ which can be found in Abramowitz and Stegun (1970).

The integrals of the fluid velocities $I_{i} = \frac{1}{{q^{2} }}\int_{{A_{i} }} {\left| {v_{0} } \right|^{2} } {\text{d}}A_{i}$ about the areas of tank wall and bottom of the nth axial mode and 1st circumferential mode of the sloshing oscillation are given by:

- Integral of the axial flow at the tank wall:

28 $$ I_{1,n}^{wall,1} = HR\int\limits_{0}^{2\pi } {\int\limits_{0}^{1} {\left| {v_{\xi } } \right|^{2} } } d\xi d\varphi = J_{1} (\lambda_{n} )^{2} \lambda_{n} \frac{\pi }{2}\left[ {\cosh (\alpha_{L} \lambda_{n} )\sinh (\alpha_{L} \lambda_{n} ) - \lambda_{n} \alpha_{L} } \right]. $$

- Integral of the radial flow at the tank bottom:

29 $$ I_{1,n}^{bottom,1} = R^{2} \int\limits_{0}^{2\pi } {\int\limits_{0}^{1} {\left| {v_{\zeta } } \right|^{2} } } \zeta {\text{d}}\zeta {\text{d}}\varphi = \frac{\pi }{2}[J_{0} (\lambda_{n} )^{2} \left[ {\lambda_{n}^{2} + 1} \right] + J_{1} (\lambda_{n} )^{2} \left[ {\lambda_{n}^{2} - 1} \right] - 1]. $$

- Integral of the flow in circumferential direction on the tank wall:

30 $$ I_{1,n}^{wall,2} = HR\int\limits_{0}^{2\pi } {\int\limits_{0}^{1} {\left| {v_{\varphi } } \right|^{2} } } d\xi d\varphi = J_{1} (\lambda_{n} )^{2} \frac{\pi }{{2\lambda_{n} }}\left[ {\cosh (\lambda_{n} \alpha_{L} )\sinh (\lambda_{n} \alpha_{L} ) + \lambda_{n} \alpha_{L} } \right]. $$

- Integral of the flow in circumferential direction on the tank bottom:

31 $$ I_{1,n}^{bottom,2} = R^{2} \int\limits_{0}^{2\pi } {\int\limits_{0}^{1} {\left| {v_{\varphi } } \right|^{2} } } \zeta {\text{d}}\zeta {\text{d}}\varphi = \frac{\pi }{2}\left[ {1 - J_{0} (\lambda_{n} )^{2} - J_{1} (\lambda_{n} )^{2} } \right]. $$

The mean value of the dissipated energy in time follows from Eq. (18) under application of the integrals of Eqs. (28–31).

32 $$ \bar{\dot{E}}_{mech,1,n} = - \frac{1}{2}\sqrt {\frac{{\mu_{L} {\varrho }_{L} \Omega }}{2}} q^{2} (I_{1,n}^{wall,1} + I_{1,n}^{wall,2} + I_{1,n}^{bottom,1} + I_{1,n}^{bottom,2} ). $$

The damping factor is given according to Eq. (17) by:

33 $$ \gamma_{1,n} (\Omega ) = \frac{1}{2R}\sqrt {\frac{{\nu_{L} \Omega }}{2}} \frac{{(I_{1,n}^{wall,1} + I_{1,n}^{wall,2} + I_{1,n}^{bottom,1} + I_{1,n}^{bottom,2} )}}{{J_{1} (\lambda_{n} )^{2} \cosh (\lambda_{n} \alpha_{L} )\sinh (\lambda_{n} \alpha_{L} )\frac{\pi }{2}\frac{{\lambda_{n}^{2} - 1}}{{\lambda_{n} }}}}. $$

Using the normalized parameter $C_{1,n}$, the Eq. (33) can be written as follows:

34 $$ \gamma_{1,n} (\Omega ) = \frac{1}{2R}\sqrt {\frac{{\nu_{L} \Omega }}{2}} C_{1,n} . $$

It is assumed that the modal damping $\kappa_{1,n}$ ratio corresponds to the damping factor $\gamma_{1,n}$ at the modal natural circular frequency $\left( {\Omega = \omega_{1,n} } \right).$ Hence, the damping ratio of the free sloshing oscillation in the nth axial and 1st circumferential mode with the natural circular frequency $\omega_{SL,n}$ is calculated using the following equation:

35 $$ \kappa_{1,n} = \frac{1}{2R}\sqrt {\frac{{\nu_{L} }}{{2\omega_{1,n} }}} C_{1,n} . $$

**Fig. 3:**
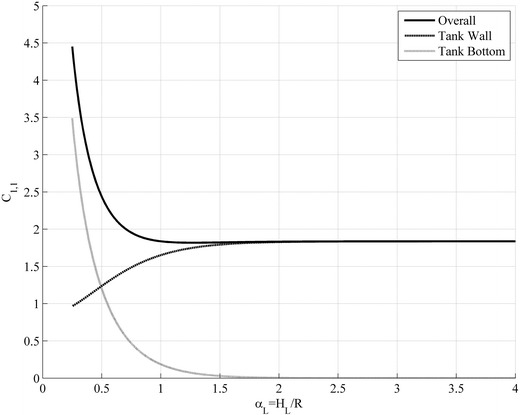
Damping parameter $C_{1,1}$ for the 1st axial and circumferential mode of the sloshing oscillation dependent on the height to radius ratio of the liquid. For broad tanks the liquid motion at tank bottom dominates the damping while for slender tanks the damping arises from the liquid motion on the upper part of the tank wall

In Fig. 3 the values of $C_{1,n}$ are shown for the 1st and 2nd axial and the 1st circumferential mode of the free sloshing oscillation in regards to the ratio $\alpha_{L} = H_{L} /R$. The parameter $C_{1,n}$ consists of contributions from the fluid friction on the tank bottom and the tank wall. Slender storage tanks have only a small part of fluid friction on the tank bottom. This results because the fluid motion is limited to the free surface of the liquid and decays rapidly into the depth of the tank.

**Fig. 4:**
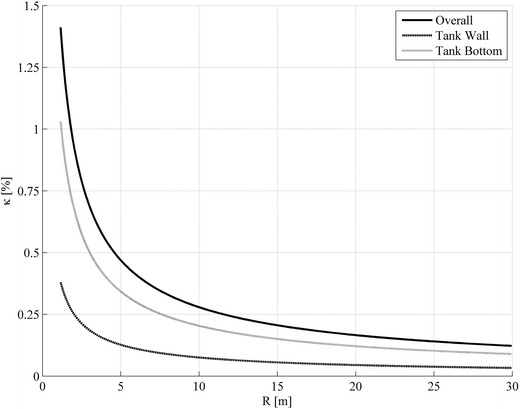
Damping ratios $\kappa$ of the free sloshing oscillation in the 1st axial and circumferential mode for a broad tank with slenderness $\alpha_{L} = 0.3$, a kinematical viscosity $\nu_{L} = 4.2 \times 10^{ - 4} {\,\text{m}}^{ 2} / {\text{s}}$ and a density $\rho_{L} = 912\;{\text{kg/m}}^{ 3}$ (oil, SAE 30 at 15.6 °C) in dependence of the tank radius R. The damping is size dependent and decays with increasing tank volume. Furthermore the liquid motion at the tank bottom dominates the damping

**Fig. 5:**
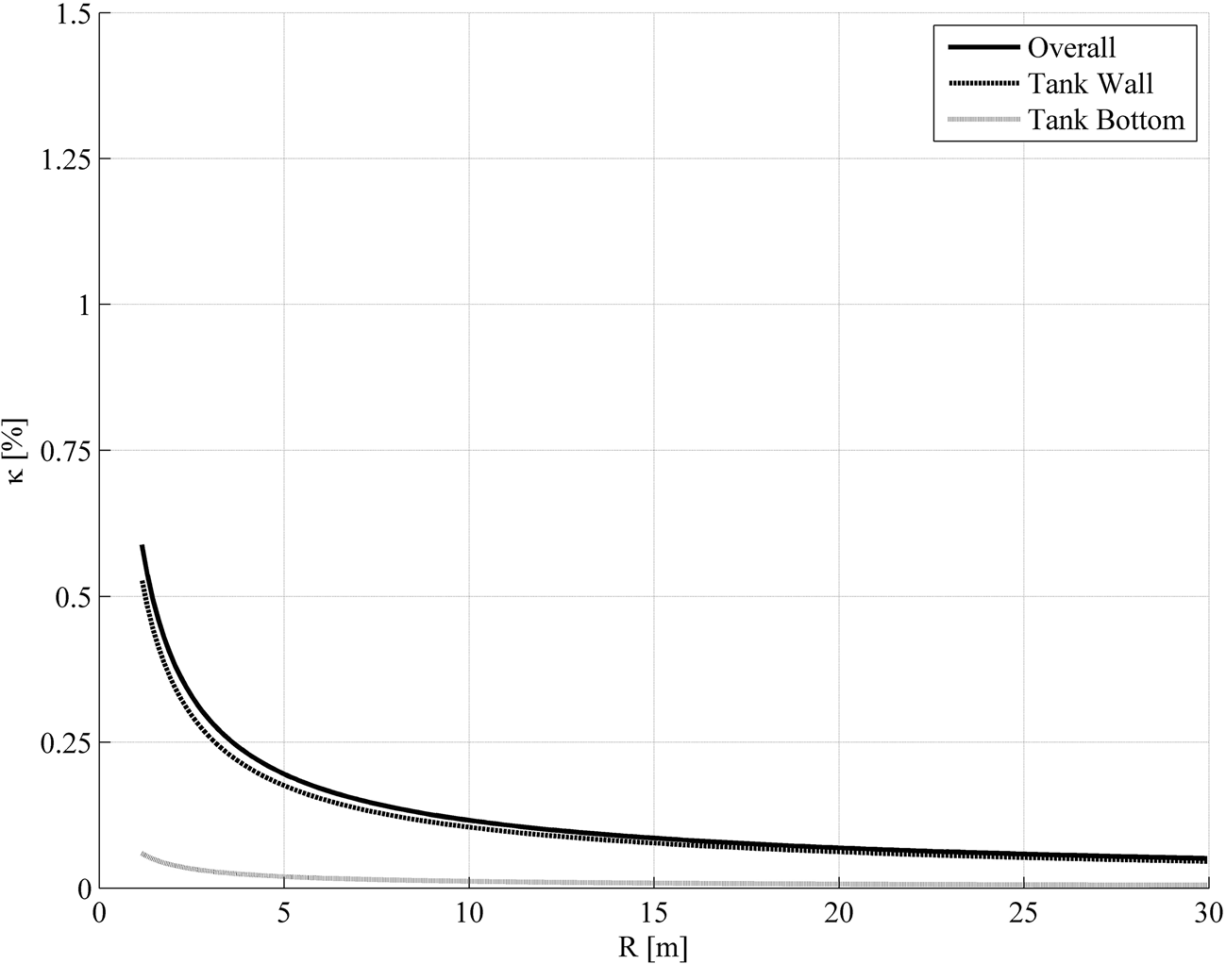
Damping ratios $\kappa$ of the free sloshing oscillation in the 1st axial and circumferential mode for a more slender tank with slenderness $\alpha_{L} = 1.0$, a kinematical viscosity $\nu_{L} = 4.2\; \times \;10^{ - 4} \;{\text{m}}^{ 2} / {\text{s}}$ and a density $\rho_{L} = 912\;{\text{kg/m}}^{ 3}$ (oil, SAE 30 at 15.6 °C) in dependence of the tank radius R. The damping is size dependent and decays with increasing tank volume. Furthermore the liquid motion at the tank wall dominates the damping

Figures 4 and 5 give the damping ratio $\kappa_{1,1}$ for variations in slenderness of the storage tank (0.3 and 1.0) and in dependence on the tank radius. From these graphs, it can be noted that the fluid damping increases exponentially if the radius of storage tanks becomes smaller. The increase of the damping results from the increase of the surface-to-volume ratio of the contained liquid ($\approx 1/R$). This is due to the fact that the transport of the impulse by diffusion increases against the impulse transport by convection.

## Conclusion

In this paper an engineering method for the determination of viscous fluid damping in liquid storage tanks has been presented. This method was applied to the sloshing motion of the free liquid surface. Damping ratios of the convective component depending on the viscosity of the fluid and the geometry of the shell were also derived. The proportion of the damping accounted for the tank wall and the tank bottom can be distinguished and examined. Concerning the damping effects of the convective component the following conclusions can be made:

- The presented distinction of fluid damping is especially important for fluids with high viscosity and if the sloshing motion has a remarkable contribution to the overall fluid response (e.g. tanks with small dimensions and high sloshing frequency or if the earthquake is dominated by low frequencies). In most practical cases of tank geometry and fluid properties a damping ratio of 0.5 % (like in Eurocode 8, Part 4) is a too optimistic assumption.
- The damping factor decreases with increasing fluid volume because of the descent of the surface-to-volume ratio of the fluid.
- The damping factor is frequency dependent; it becomes smaller for higher sloshing modes.

It would also possible to apply the method also to the impulsive pressure component, but the impulsive component is dominated by the structural damping of the tank shell. Using normalized damping coefficients, an easy-to-use procedure for the calculation of the damping ratios is proposed and it seems to be applicable for seismic codes. It would be interesting and maybe a part of a future work to compare the predicted damping ratios with experimental results and those of more sophisticated numerical calculations.
